# Evaluation of ethanol-based extraction conditions of sorghum bran bioactive compounds with downstream anti-proliferative properties in human cancer cells^☆^

**DOI:** 10.1016/j.heliyon.2019.e01589

**Published:** 2019-05-07

**Authors:** Sarah Cox, Leela Noronha, Thomas Herald, Scott Bean, Seong-Ho Lee, Ramasamy Perumal, Weiqun Wang, Dmitriy Smolensky

**Affiliations:** a Grain Quality and Structure Research Unit, Agricultural Research Service, U.S. Department of Agriculture, Manhattan, KS, USA; b Arthropod-Borne Animal Disease Research Unit, Agricultural Research Service, U.S. Department of Agriculture, Manhattan, KS, USA; c Department of Nutrition and Food Science, University of Maryland, College Park, MD, USA; d Department of Agronomy, Kansas State University, Manhattan, KS, USA; e Department of Food Nutrition Dietetics and Health, Kansas State University, Manhattan, KS, USA

**Keywords:** Cell biology, Food science, Natural product chemistry, HP, High phenolic black sorghum bran PI570481, CB, Commercial black sorghum bran, CS, Commercial sumac sorghum bran, GT, Matcha green tea powder

## Abstract

Certain foods such as turmeric and green tea have been extensively studied for anticancer properties, while high polyphenol sorghum has not received the same attention. Some bioactive compounds in *Sorghum bicolor* with anticancer activity have been identified, indicating the further need for research and screening methods of high polyphenol sorghum varieties. This study was aimed at improving the extraction of sorghum bioactive compounds by using food-grade solvents using ethanol and citric acid. We used three sorghum varieties and green tea (GT) as a control. The extraction methods were screened for anti-proliferative properties in HepG2 and HCT-15 cancer cell lines, using a cell viability assay. Extraction conditions were improved for anti-proliferative compounds from a high-phenolic sorghum variety (HP), sumac sorghum (CS), and GT. HP was more effective at inhibiting cell viability than CB, CS, and GT. The results demonstrate an efficient method for extracting sorghum bioactive compounds for future anticancer research using food approved ingredients.

## 1 Introduction

Cancer is one of the leading causes of death in the United States and the rest of the world, and the possibility of cancer prevention through dietary interventions, especially with high-polyphenol foods, has become an increasingly attractive research area (Duthie et al., 2000). *Sorghum bicolor* is a genetically diverse crop and several varieties (germplasms) exist with extremely high amounts of polyphenols (Harrison, 2015). Previous research suggests that high-polyphenol sorghum may have components with a strong anticancer activity (Awika and Rooney, 2004; Smolensky et al., 2018; Yang et al., 2009). To further evaluate sorghum polyphenols as anticancer agents and potentially market sorghum and/or its extracts as health-promoting supplements, the identification of both the optimal extraction methods and specific anticancer molecules are crucial. In many previous studies, methanol, acetone, and/or hydrochloric acid have been used to extract sorghum polyphenols (Awika et al., 2005; Devi et al., 2011). However, these extraction methods present problems when evaluating sorghum polyphenols for future in vivo studies and the potential marketing of the extracts. Methanol is not an approved food ingredient and both acetone and hydrochloric acid are highly regulated and their application is limited to very specific food processes with only very small residual amounts allowed. On the other hand, both ethanol and citric acid are generally recognized as safe (GRAS) (Food additive status list, 2018). Both ethanol and citric acid have been successfully used in the extraction of polyphenols from green tea (Rusak et al., 2008). Furthermore, previous research has suggested that ethanol-based extractions of sorghum polyphenols are absorbable in the intestinal tract, while no such data exist for other extraction methods (Jimenez-Ramsey et al., 1994). Sorghum bioactive compounds extracted using 50% v/v ethanol were previously used in cell culture studies (Burdette et al., 2010). Our laboratory previously used 50% v/v ethanol extracts of high-polyphenol sorghum bran to evaluate potential anticancer effects of sorghum in HepG2 and Caco2 cell lines (Smolensky et al., 2018). While extraction with 50% v/v ethanol provided positive results, the extraction method used should be further improved for future research. Previous research has suggested that the addition of citric acid and/or an increase in the extraction temperature can enhance the extraction of health promoting polyphenols from plants such as green tea and turmeric (Paulucci et al., 2013; Rusak et al., 2008; Zimmermann and Gleichenhagen, 2011).

In order to develop the optimum extraction procedure, which could be further used in anticancer research including in vivo studies, we investigated various phenolic extraction procedures by adjusting the ethanol content, adding citric acid, and increasing heat during the extraction. Studies evaluating extraction methods of bioactive compounds from plant tissue tend to rely on chemical assays exclusively. However, to our knowledge, assessments of the biological effects of these extraction conditions have not been conducted. Although chemical assays provide some useful information regarding the content of the extracts, measurement of the further downstream effects of various extraction conditions is also important. In addition, the results of chemical assays may show little relevance for the further downstream biological effects when the complete chemical makeup of the crude extracts is unknown. In order to test the biological effects of the extraction conditions, the anticancer effects related to the extraction conditions were measured using the MTS cell viability assay with two cancer cell lines, HCT-15 and HepG2. This research will also provide better material for compound identification by identifying the most effective crude extraction method using food-grade solvents.

## 2 Materials and methods

### 2.1 Reagents

The chemicals and consumables used were purchased from Fisher Scientific (Pittsburgh, PA, USA), unless otherwise stated.

### 2.2 Plant material

Three types of sorghum bran were used to evaluate extraction procedures. Two of these were commercial sorghum varieties grown in western Kansas, namely, commercial black sorghum (TX430) bran (CB) and commercial sumac sorghum bran (CS). The third type was the novel high-polyphenol black sorghum (HP; accession number, PI570481), which has been previously used in our studies (Smolensky et al., 2018); this variety was grown in Puerto Vallarta, Mexico, during the 2014 winter nursery season and the bran was decorticated in house. Organically grown matcha green tea (Jade Leaf brand; GT) was purchased commercially.

### 2.3 Total phenolic extraction

The dry material was combined with solvents A-F (10% w/v; Table 1), and the samples were allowed to mix on a shaker for 2 h at 20 °C and stored at −20 °C overnight. The samples were then centrifuged at 3000 × *g* for 10 min, and the solid pellet was discarded. The supernatant was used as the total phenolic extract.

**Table 1:** Composition of the solvents used to extract bioactive compounds from sorghum bran.

Solvent	Ethanol % v/v	Citric acid % w/v
A	50.00%	0.00%
B	70.00%	0.00%
C	90.00%	0.00%
D	50.00%	5.00%
E	70.00%	5.00%
F	90.00%	5.00%

In order to test the effects of increasing temperature on total phenolic extraction, Solvent E (70% v/v ethanol plus 5% w/v citric acid) was chosen as a solvent due to its efficacy, and total phenolic extraction was performed using the protocol stated above, with the temperature during the 2-h shaking period being adjusted to 20 °C, 40 °C, or 60 °C.

### 2.4 Measurement of total phenolic content

The previously published Folin-Ciocalteu (FC) assay was used to measure the total phenolic content in the total phenolic extracts (Herald et al., 2012). Gallic acid 0–800 μg/L was used as the standard to determine the gallic acid equivalent per gram (GAE/g) levels of the dry material. The diluted sample (25 μL) was combined with 75 μL of distilled water and 25 μL of FC reagent and incubated for 6 min at 20 °C. Next, 100 μL of 7.5% w/v Na_2_CO_3_ was added to each well, and the plate was incubated at 20 °C in the dark for 90 min. After the incubation period, absorbance was measured using a Biotek H4 Plate Reader (Winooski, VT, USA) at 765 nm.

### 2.5 Cell culture

Human colorectal adenocarcinoma (HCT-15) and human hepatocellular carcinoma (HEPG2) cells were purchased from American Type Culture Collection (Manassas, VA). HCT-15 cells were grown in RPMI 1640 medium, and HepG2 cells were grown in minimum essential medium (MEM). The media were supplemented with 10% fetal bovine serum and 1X anti-biotic anti-mycotic solution. The cells were grown and treated at 37 °C and 5% CO_2_.

### 2.6 Cell viability assay

Cell viability was measured using the Promega CellTiter 96® Aqueous One Solution Cell Proliferation Assay (Madison, WI) in accordance with the kit instructions. In brief, 5 × 10^3^ cells were plated in a 96-well tissue culture plate and allowed to attach for 24 h. The cells were then treated with either the extract or the specific solvent used in the extraction vehicle control, after which they were allowed to grow for an additional 48 h. The cells were then washed with phosphate-buffered saline (PBS), and 100 μL of fresh media containing 20% v/v MTS reagent was added to the wells. The plates were incubated for 45 min, and absorbance was measured at 490 nm on a Biotek H4 Plate Reader (Winooski, VT, USA). All data (minus blank) were normalized to the values for the specific solvent (vehicle) treatment.

### 2.7 Statistical analysis

Statistical analyses were performed using two-way analysis of variance (ANOVA) followed by Tukey's test for multiple comparisons in GraphPad Prism version 7.03 for Windows (GraphPad Software, La Jolla, CA, USA: www.graphpad.com).

## 3 Results

### 3.1 Solvent composition effects on the total phenolic content and anti-proliferative effects in cancer cells of the sorghum bran extracts

**Fig. 1:**
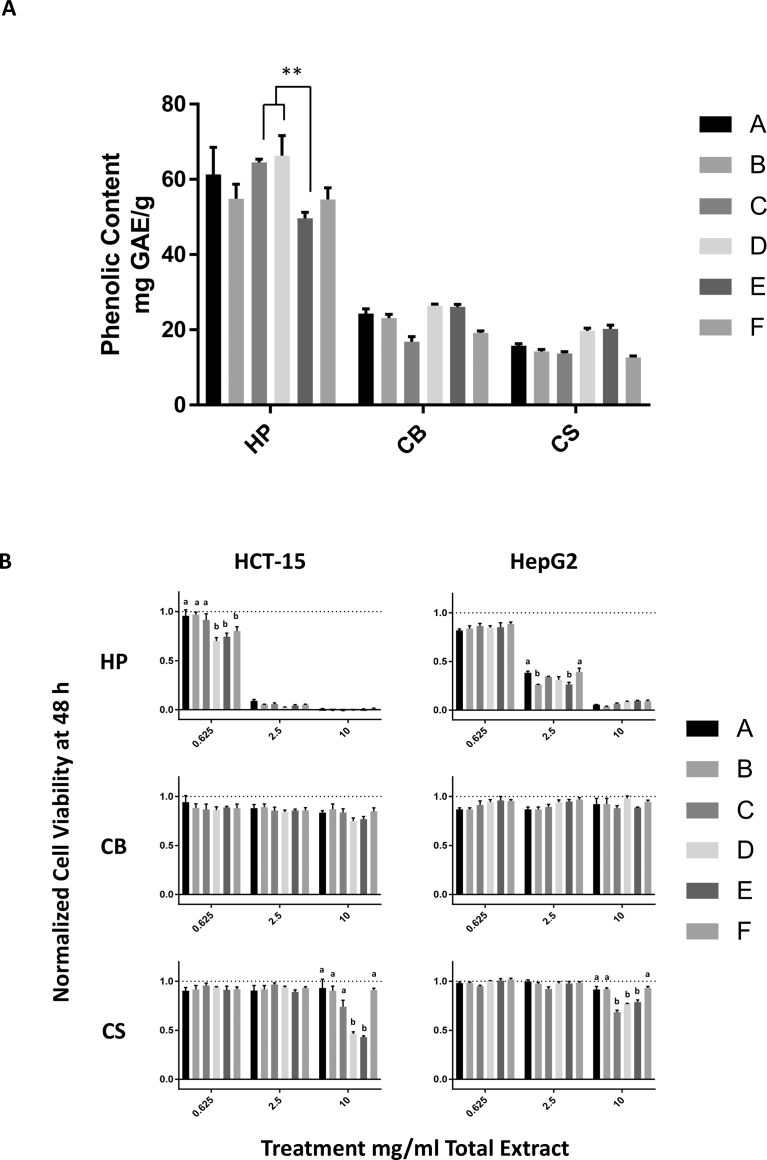
Effects of ethanol content and citric acid on extraction of sorghum bioactive compounds. A) Total phenolic content, extracted using solvents A-F, of sorghum bran extracts represented as mg gallic acid equivalents obtained from 1 g of dry bran (mg GAE/g). Data represent the averages of three separate extractions ± SEM **P ≤ 0.01. B) Effect of the three sorghum bran extracts, obtained using solvents A-F, on cancer (HCT-15 left and HepG2 right) cell viability after 48 h of treatment. The data represent the average of three separate extractions ± SEM. Different letters represent a significant difference between solvents used with the same bran and at the same dose with P ≤ 0.05.

The total phenolic content varied with different solvents (Fig. 1A). For HP, the total phenolic content ranged from 49.6 mg GAE/g (solvent E) to 66.3 mg GAE/g (solvent D). For CB, the total phenolic content ranged from 17.0 mg GAE/g (solvent C) to 26.6 mg GAE/g (solvent D), whereas for CS, it ranged from 12.6 mg GAE/g (solvent F) to 20.2 mg GAE/g (solvent E). The only significant difference in the total phenolic content was observed in HP extractions with solvent E (49.6 mg GAE/g) in comparison with those with solvents C and D (64.5 mg GAE/g and 66.3 mg GAE/g, respectively). There was no significant difference in the total phenolic content extracted from CB and CS.

The bioactivity of compounds obtained using various solvents was assessed with the MTS assay, which measured the inhibition of cancer cell proliferation/viability in two different cancer cell lines, HCT-15 and HepG2 (Fig. 1B). For extracts obtained using HP, both 2.5 mg/mL and 10 mg/mL doses resulted in almost complete reduction of viability in HCT-15 cells and therefore did not show significant differences among solvents. However, at a dose of 0.625 mg/mL, HP had varying effects on cell viability in HCT-15 cells; the extracts obtained using solvents D and E reduced HCT-15 cell viability to a significantly greater degree than those obtained using solvents A-C. In assessments with HepG2 cells and HP extracts, the 0.625 mg/mL dose resulted in a modest reduction in cell viability with no differences observed between extracts, while the 10 mg/mL dose resulted in almost complete reduction in cell viability with no significant differences between extracts either. However, at a dose of 2.5 mg/mL, HP extracts had varying effects on cell viability, with the extracts obtained using solvents B and E showing a significantly greater anti-proliferative effect than those shown by the extracts obtained using solvents A and F.

Treatments performed using CB extracts showed only a modest reduction in cell viability at all doses for both HCT-15 and HepG2 cells, with no significant differences in the cell viability-reducing ability between extracts obtained with different solvents. In contrast, the CS extracts did not show significant cell viability-reducing ability at a dose of 0.625 mg/mL or 2.5 mg/mL in both HCT-15 and HepG2 cells. However, at the 10 mg/mL dose, the CS extracts showed significant anti-proliferative effects that differed according to the solvents used: CS extracts obtained using solvents D and E showed a significantly greater anti-proliferative effect than that shown by the extracts obtained using solvents A, B, C, and F in HCT-15 cells while extracts obtained using solvents C, D, and E showed a significantly greater anti-proliferative effect than that shown by the extracts obtained using solvents A, B, and F in HepG2 cells.

Overall, solvent E (70% v/v ethanol with 5% w/v citric acid) was the most effective in extracting bioactive compounds with anti-proliferative effects on both cancer cell lines for both HP and CS. Therefore, solvent E was chosen to evaluate the effects of increasing temperature on the extraction of bioactive compounds with anti-proliferative effects on cancer cells.

### 3.2 Temperature effects bioactive compounds extraction and down-stream anti-proliferative properties in HCT-15 cancer cells

**Fig. 2:**
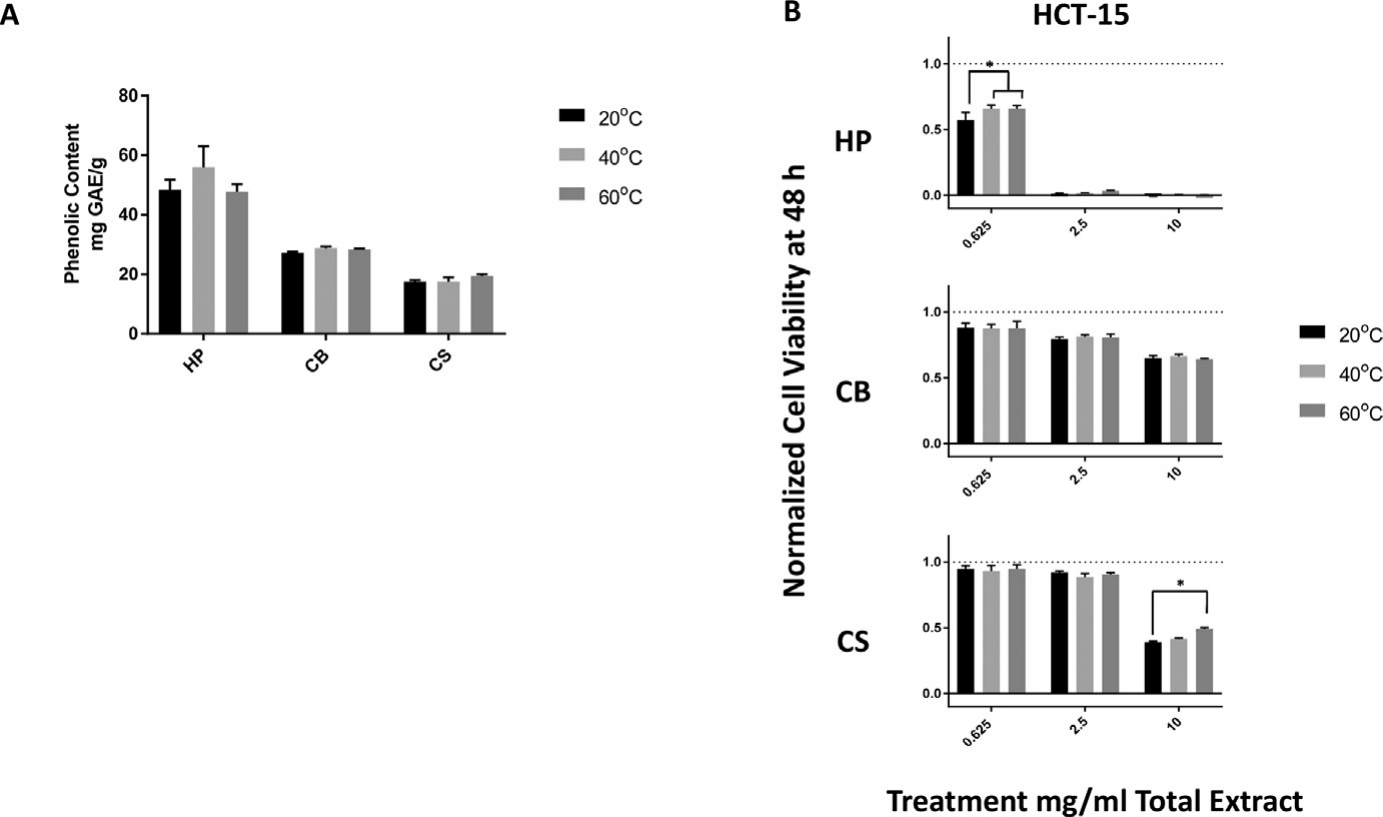
Effects of increasing temperature on extraction of sorghum bioactive compounds by using solvent E. A) Total phenolic content, extracted using three temperatures, of sorghum bran extracts represented as mg gallic acid equivalents obtained from 1 g of dry bran (mg GAE/g). B) Effect of the three sorghum bran extracts, extracted at 20 °C, 40 °C, and 60 °C on HCT-15 cancer cell viability after 48 h of treatment. The data represent the anti-proliferative effects of three separate extracts ± SEM. Different letters represent significant differences between extraction temperatures used with the same bran and at the same dose with *P ≤ 0.05.

Temperature did not have a significant effect on the extraction levels of total phenolic content as measured by the FC assay (Fig. 2A). However, an increase in temperature did cause variations in the anti-proliferative effects of both HP and CS extracts (Fig. 2B). An increase in the extraction temperature from 20 °C to either 40 °C or 60 °C significantly reduced the efficacy of the HP extract in reducing HCT-15 cell viability at a dose of 0.625 mg/mL. In contrast, while CB extracts showed no temperature-related differences in their effect on HCT-15 cell viability, CS extracts obtained at 60 °C showed significantly reduced anti-proliferative effects on HCT-15 cells in comparison with those of CS extracts obtained at 20 °C. Since the increasing temperature did not improve the extraction of the total phenolic content and in fact adversely affected the ability of the extracts to reduce the viability of HCT-15 cells, extractions at higher temperatures were not evaluated further.

### 3.3 Efficacy of sorghum bran extracts compared to green tea extracts

Green tea has been extensively studied both in vitro and in vivo for its anticancer effects (Hayakawa et al., 2016; Ullah et al., 2016). Therefore, we compared the effects of various extraction conditions on the anti-proliferative effects of green tea extracts on both HepG2 and HCT-15 cancer cells. GT is considered to be particularly high in polyphenol content and was chosen because it is available in powdered form and can be easily incorporated into foods, similar to sorghum bran (Phongnarisorn et al., 2018).

**Fig. 3:**
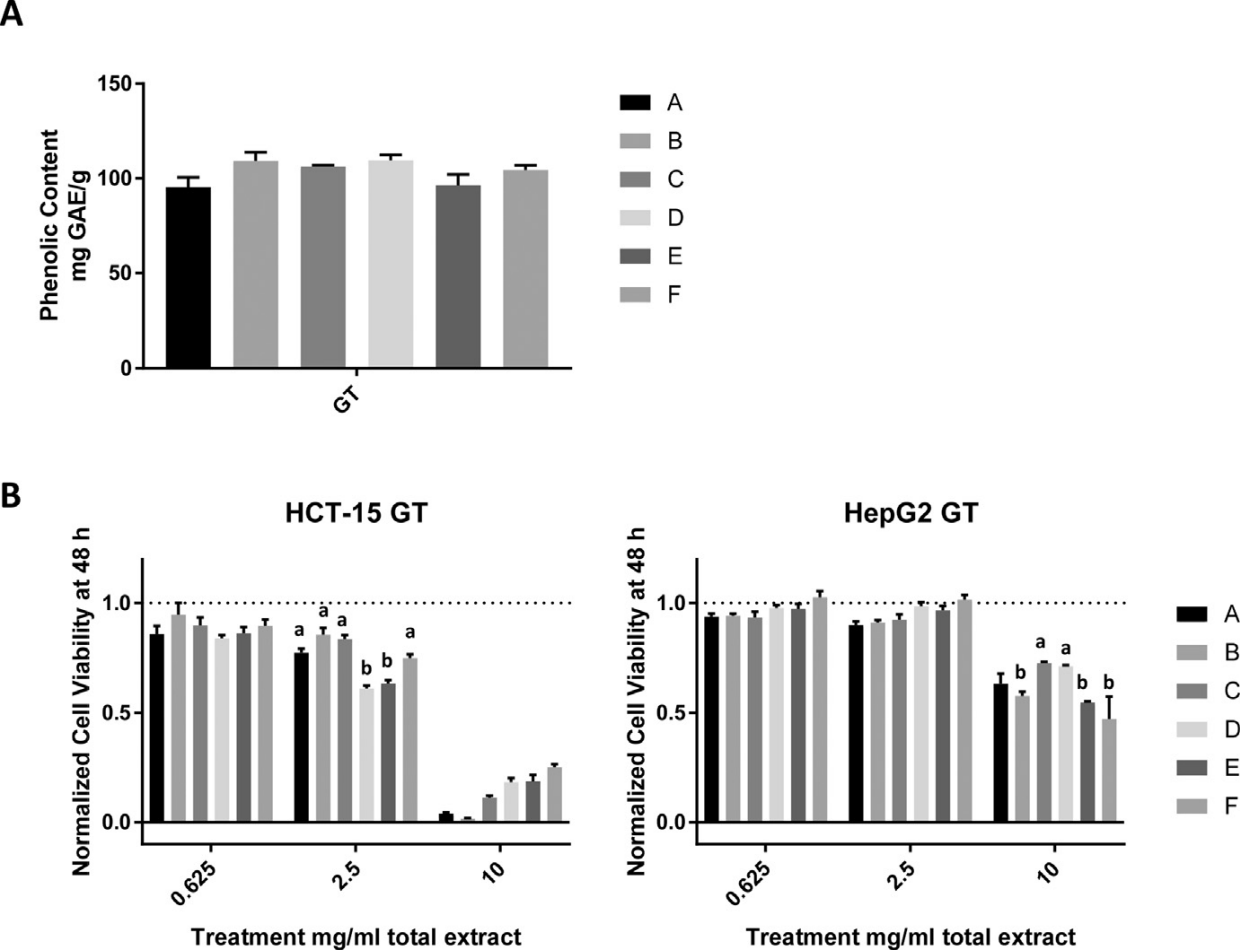
Effects of ethanol content and citric acid on extraction of green tea bioactive compounds. A) Total phenolic content of matcha green tea extracts obtained using solvents A-F, represented as mg gallic acid equivalents extracted from 1 g of matcha green tea powder (mg GAE/g). B) Cell viability of HCT-15 cells (left) and HepG2 cells (right) after 48 h of treatment. The data represent the anti-proliferative effects of three separate extracts ± SEM. Different letters represent significant differences between extraction temperatures used with the same bran and at the same dose with *P ≤ 0.05. Note: No viable HCT-15 cells were visible at 10 mg/mL treatments with all solvents and color changes in media were observed when citric acid was present, indicating that the results may not represent actual cell viability of HCT-15 cells for that high dose.

**Fig. 4:**
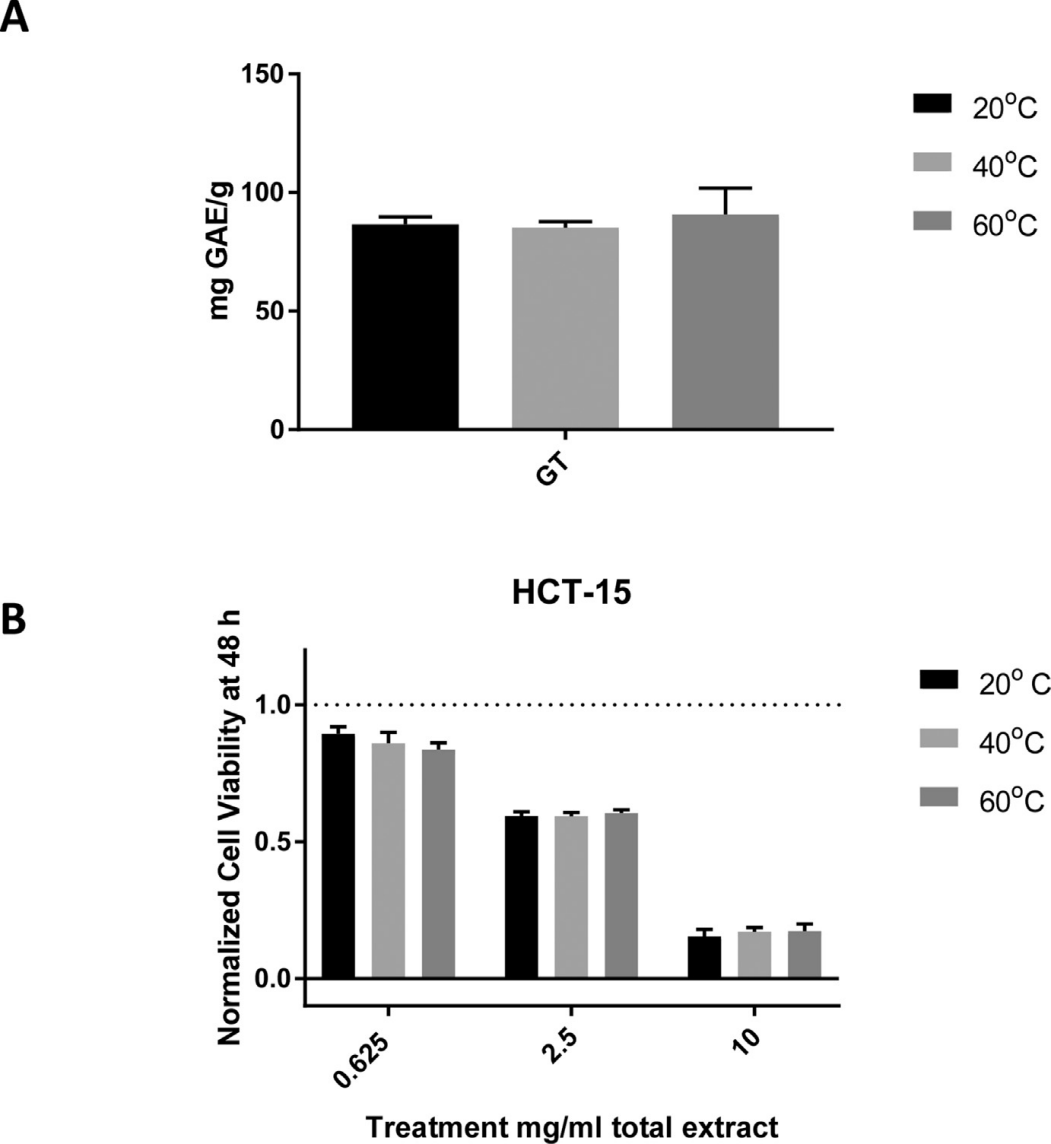
A) Effects of increasing temperature on extraction of green tea bioactive compounds obtained using solvent E, represented as mg gallic acid equivalents extracted from 1 g of matcha green tea (mg GAE/g). B) Cell viability of HCT-15 cells after 48 h of treatment using green tea solvents extracted with solvent E at 20 °C, 40 °C, and 60 °C. No significant differences in phenolic contents or effects on HCT-15 cell viability were observed. The data represent the average of three separate extractions ± SEM.

We used the six solvent conditions A-F to extract GT bioactive compounds and measured the total phenolic levels and the extracts' ability to inhibit cancer cell proliferation. While the different solvents did not yield significant differences in total phenolic levels (Fig. 3A), similar to the findings for sorghum extracts, the extract obtained with solvent E (70% v/v ethanol with 5% w/v citric acid) was the most effective at inhibiting cancer cell proliferation in HepG2 and HCT-15 cells (Fig. 3B). The effects of different extraction temperatures were also tested on the GT extracts by using solvent E, and no significant differences were observed in the total phenolic content extracted or the efficacy against cancer cells (Fig. 4A and B).

**Fig. 5:**
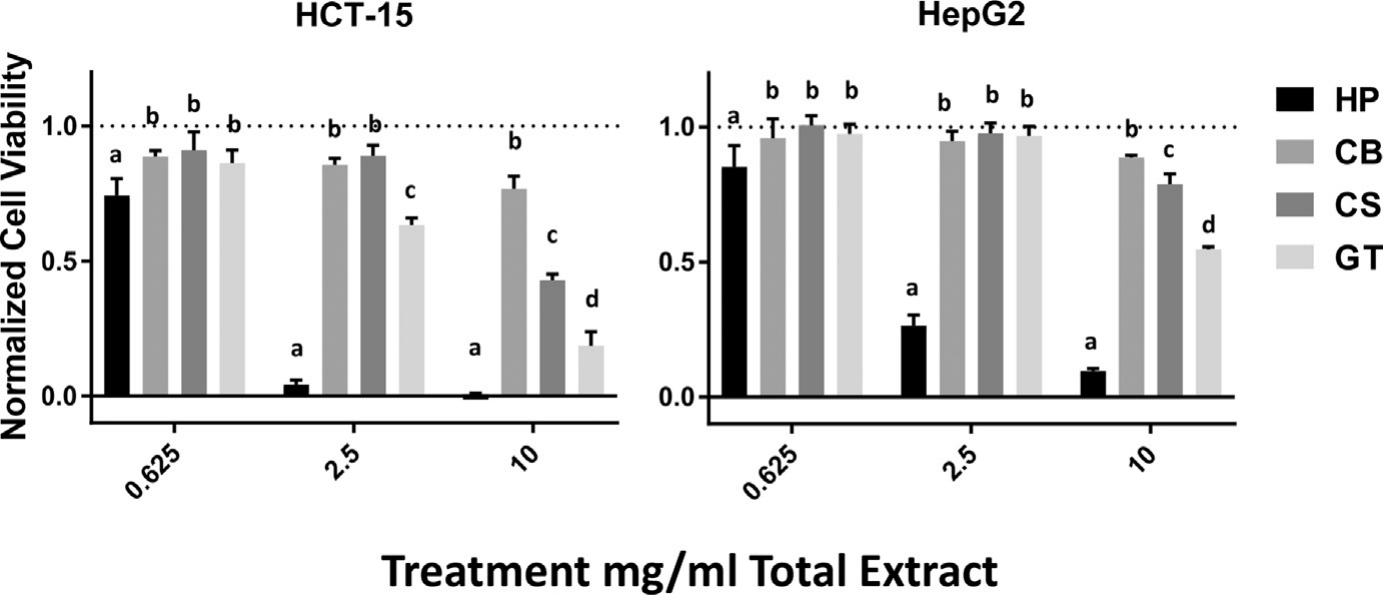
Direct comparison of the anti-proliferative effects of sorghum bran and green tea bioactive compounds extracted under identical conditions (solvent E at 20 °C). HCT-15 (left) and HepG2 (right) cells. The data represent the average of three separate extracts ± SEM. Different letters represent a significant difference between HP, CB, CS, and GT at the same treatment dose, P ≤ 0.05. t

In a direct comparison of the anti-proliferative effects of the three sorghum bran extracts against the GT extract obtained using the same extraction conditions (solvent E, 20 °C), the HP extract was significantly more effective in inhibiting HCT-15 and HepG2 cancer cell proliferation than were the extracts of the other two commercial varieties of sorghum and commercial green tea, at all three doses tested (Fig. 5). The GT extract inhibited cancer cell proliferation more effectively than both the CB and CS extracts at doses of 2.5 mg/mL and 10 mg/mL for HCT-15 cells and at the dose of 10 mg/mL for HepG2 cells. The CS extract was significantly more effective than the CB extract at inhibiting the proliferation of both HCT-15 and HepG2 cells at a dose of 10 mg/mL.

## 4 Discussion

The objectives of this study were to evaluate and improve the extraction conditions of sorghum bioactive compounds with potential antiproliferative properties against cancer cells and to compare the antiproliferative effects of HP, CS, CB, and commercial GT, in vitro. Our results showed that varying the concentration of ethanol and adding citric acid produced extracts with similar amounts of polyphenols. There was no significant difference observed between the phenolic content of the extracts with the exception of less phenolic content in HP extracts obtained using solvent E when compared to that with solvent C and D. Interestingly, HP and CS extracts obtained using solvents D and E had the greatest anti-proliferative effects on both cancer cells lines. While extracts obtained using solvents D and E showed similar results in the cell viability assay, those obtained using solvent E has a significantly lower polyphenol content than that in the extracts obtained using solvent D, indicating the bioactive compounds obtained with solvent E had a higher anti-proliferative. These results raise three possibilities. Solvent E aids in the extraction of unique bioactive compounds (not limited to polyphenols) with greater anti-proliferative bioactivity by 1) facilitating the extraction of specific molecules that have a greater anti-proliferative effect on cancer cells, 2) facilitating the extraction of the same bioactive compounds as those obtained using other solvents, but allowing them for to be more readily taken up by cells due to a cleaner extraction, and 3) chemically modifying the bioactive compounds to have a greater anti-proliferative effect on cancer cells. Sorghum has been shown to contain several specific compounds with potential anticancer properties. 3-Deoxyanthocyanins in black sorghum have been shown to possess both anticancer and antioxidant properties in vitro (Yang et al., 2009). Aside from 3-deoxyanthocyanins, sorghum contains flavones with estrogenic properties, which have an anticancer effect in vitro (Yang et al., 2012). Differences were observed between the effects of solvents on two different cancer cell lines. For example, 90% ethanol extract of CS was effective in reducing the viability of HepG2 cells but not HCT-15 cells, compared to the extracts obtained using other solvents. This further demonstrates the need for screening extraction methods using biological systems of interest.

In conclusion, the results indicate that using solvent E (70% v/v ethanol, 5% w/v citric acid) was more effective for the extraction of bioactive compounds with potential antiproliferative effects against cancer cells from HP, CS, and commercial GT than was solvent A (50% v/v ethanol), as published previously. Our findings stress that chemical assays, which estimate the total amount of phenolic content, may not correlate with the biological effects of crude extracts from sorghum and other plant material. This is especially true when the exact composition of the crude extract is unknown. Because biological effects can vary between different sorghum varieties, screening methods for studying plant extracts should not only involve chemical assays but evaluations of biological effects of interest as well. Future research should focus on identifying the specific antiproliferative compounds in sorghum bran that can be screened against existing cancer models both in vitro and in vivo. The ability to extract these compounds more efficiently in a solvent containing food approved ingredients will greatly contribute to future studies.

## Declarations

### Author contribution statement

Sarah Cox: Conceived and designed the experiments; Performed the experiments; Analyzed and interpreted the data; Wrote the paper.

Leela Noronha, Weiqun Wang, Seong-Ho Lee: Conceived and designed the experiments; Analyzed and interpreted the data; Wrote the paper.

Thomas Herald, Scott Bean: Conceived and designed the experiments; Analyzed and interpreted the data; Contributed reagents, materials, analysis tools or data; Wrote the paper.

Ramasamy Perumal: Analyzed and interpreted the data; Contributed reagents, materials, analysis tools or data; Wrote the paper.

Dmitriy Smolensky: Conceived and designed the experiments; Performed the experiments; Analyzed and interpreted the data; Contributed reagents, materials, analysis tools or data; Wrote the paper.

### Funding statement

This work was supported by the United States Department of Agriculture, Agricultural Research Service. This paper is Contribution No. 19-257-J from the Kansas Agricultural Experiment Station.

### Competing interest statement

The authors declare no conflict of interest.

### Additional information

No additional information is available for this paper.
